# Breastfeeding and the Risk of Infant Illness in Asia: A Review

**DOI:** 10.3390/ijerph17010186

**Published:** 2019-12-26

**Authors:** Mi Kyung Lee, Colin Binns

**Affiliations:** 1 College of Science, Health, Engineering and Education, Murdoch University, Murdoch, WA 6150, Australia; 2 School of Public Health, Curtin University, Bentley, WA 6102, Australia

**Keywords:** breastfeeding, infection, Asia, review

## Abstract

Infancy remains the most vulnerable period of human life for death, illness, and establishing a lifetime trajectory of growth and health. It is estimated that there are 5.3 million deaths under five years of age worldwide and approximately 800,000 lives could be saved by improving breastfeeding rates and duration. In Asia, an estimated 300,000–350,000 child deaths could be prevented with optimal breastfeeding and the majority would be under 12 months of age. We present a systematic review of studies of infection and breastfeeding in infants in Asia and further review interactions of selected infectious diseases and breastfeeding. Initially, 2459 records of possible interest were identified, 153 full text papers were reviewed in detail, and 13 papers describing diarrhoeal disease and/or acute respiratory tract infection were selected for inclusion in the review. Additional papers were selected to discuss specific diseases and their relationship to breastfeeding. The review found that a variety of methods were used with differing definitions of breastfeeding and diseases. Overall, breastfeeding when compared to the use of infant formula, is associated with significantly lower rates of diarrhoeal disease and lower respiratory tract infection, with a reduction of 50% or more to be expected, especially in infants under six months of age. The relationship between breastfeeding and specific diseases including measles and HTLV1 were reviewed. Breastfeeding reduces some disease rates, but there remain a few conditions where breastfeeding may be contra-indicated.

## 1. Introduction

The importance of nutrition for health has been acknowledged for millennia, since the days of Hippocrates [1]. However it is only in the last six decades that any significance has been given to the relationship between nutrition and infection. Understanding this relationship and implementing nutrition promotion programs has been instrumental in the substantial improvements in infant and child health made in the last few decades. In China, the largest nation in the region (and the world), in the past 50 years the infant mortality rate has fallen from 84 in 1000 live births to the current level of seven per 1000, a public health story demonstrating remarkable progress [2,3]. Widespread recognition of the importance of this relationship followed the publication of the landmark WHO monograph on the interactions of nutrition and infection in 1968 [4]. Scrimshaw has summarized the history of the development of this concept in the 20th century [5].

The association between breastfeeding and infection was studied in the 1950s and Scrimshaw includes a description of a survey completed in the Punjab by Hartemann in 1961 [4]. In this study the infant mortality rate was eight times greater in infants “artificially” fed from birth compared to those who were breastfed. Beginning in the late 1950s, the Institute for Nutrition of Central America and Panama (INCAP) sponsored studies in Guatemala which added further supporting evidence to this concept [6].

The lack of randomized control trials of breastfeeding means that studies of breastfeeding reach rarely the highest standards of evidence required in systematic reviews. The major difficulty in undertaking a review of breastfeeding is that it is unethical to undertake randomized controls. The known advantages of breastmilk make it impossible to randomize infants into a control group [7]. The Promotion of Breastfeeding Intervention Trial PROBIT trial is the closest that we have to the large randomized controlled trials that are usually found in systematic reviews of pharmaceutical therapies. The PROBIT study was a cluster-randomized controlled trial of a health promotion intervention to promote exclusive breastfeeding. It commenced in Belarus in 1996 with the recruitment of 17,046 mother–infant dyads [8]. The hospitals were randomized to receive a health education training program based on the baby-friendly hospital initiative (BFHI), which was designed to increase breastfeeding duration and the proportion of mothers breastfeeding exclusively. However as recruitment did not commence until the first month postpartum, only mothers who had initiated breastfeeding were enrolled, and some of these would have already received some infant formula and hence were misclassified as exclusively breastfed as shown in the study by Mathias et al. [9]. There was no control group of formula fed infants and hence it was a cluster trial of health promotion to extend breastfeeding duration [10,11]. Despite limitations, the study has been very useful and has shown, for example, the value of the BFHI in prolonging breastfeeding duration, the role of breastfeeding in reducing gastrointestinal infections, and that cognitive development is related to breastfeeding quantity and quality [8]. There are no similar large-scale studies available in Asia for comparison with PROBIT. Because if this limitation, studies on the importance of breastfeeding rely on the accumulation of observational studies.

Conclusions on the benefits of breastfeeding for protection against infection have relied on the accumulation of studies mostly from South America and the African continent, with a limited number of studies from Europe and other higher income regions. Typical of observational studies of breastfeeding and infection is the cohort study of 4164 in the Netherlands where breastfeeding was associated with a reduction in morbidity and mortality in children less than five years of age, particularly those exclusively breastfed up to four and six months of age [12]. There have been no recent reviews of studies of breastfeeding and infection specifically in Asia. 

The aim of this study was to undertake a systematic review of breastfeeding and infection in Asia. Studies of acute respiratory infections and diarrhoeal disease with adequate descriptions of breastfeeding definitions were tabulation. Information was compiled on other infant infections and breastfeeding.

## 2. Materials and Methods

**Figure 1:**
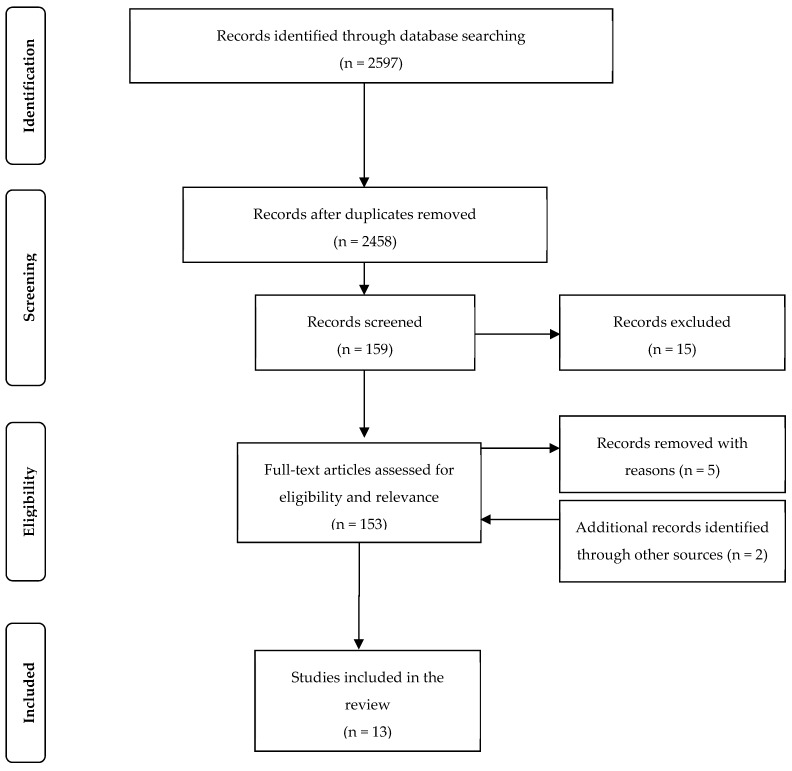
PRISMA diagram of selection of studies reviewed in this paper.

The Preferred Reporting Items for Systematic Reviews and Meta-Analyses (PRISMA) protocol was followed for this review and the search strategy results are shown in Figure 1 [13]. 

Keywords used for searching were: breastfeeding, infant feeding, infant formula, illness, or infection. These keywords were combined with Asia or China or Korea or Japan or Taiwan or Laos or Cambodia or Vietnam or Philippines or Malaysia or Singapore or Myanmar or Indonesia or Papua New Guinea or India or Bangladesh or Pakistan or Maldives.

The following comprehensive English-language databases were used to search for studies: Web of Knowledge, PubMed, Science Direct, and Proquest. Searches were limited to infants (less than 12 months old). Papers published 1990 or later were included. A sample size of a minimum 200 was needed for inclusion in the review. For example, one classic study from Indonesia had a sample of only 33 infants and was therefore excluded [14]. 

A total of 166 papers were identified that met the inclusion criteria, and this was reduced to 140 after duplicates were removed. The quality assessment of each study included an assessment of the definitions of breastfeeding used, the size and selection of the sample, and the length of the recall time to the feeding mode and morbidity. The ethical difficulties of research into breastfeeding, only observational studies, means that no studies were of the highest quality rating. The types of study: cross-sectional, cohort, or case-control are listed in Table 1.

A number of studies were excluded due to a lack of standardised definitions or measurements or long recall periods. Information about breastfeeding is more accurate if it is recorded in a cohort study with a time interval as short as practical. Examples of studies excluded are given here to indicated the type of shortcomings encountered in published research studies on breastfeeding:

In a study in Japan, mothers were interviewed when their infants were admitted to hospital. The sample was aged between 6 and 18 months and mothers were asked to recall feeding methods soon after birth. In this study the recall period was too long and the variation in the infants age created further uncertainty in the recall length [15].

In a 4-year prospective study to determine risk factors for severe community acquired pneumonia in children in Southern China breastfeeding was measured as a binomial variable, yes or no. In this study 100% were classified as “breastfeeding” and hence no effect of infant feeding method could be measured [16]. This study ignored the effect that the introduction of formula feeds at an early age can have on immune responses and the composition of the microbiome.

A cross-sectional study in India of 3671 children under 5 years of age. Breastfeeding was not measured adequately as mothers were retrospectively questioned up until five years after the event and no definition of exclusive breastfeeding was given in the paper [17]. 

A study by Perrera in Sri Lanka included 285 cases and 58 controls. Mothers were interviewed to determine how long the infants/children had been exclusively breastfed. The authors concluded that EBF infants had lower odds of developing diarrhoeal or respiratory disease [18]. However, the age at interview ranged from 6 months to 12 years, resulting in a varying length of recall with the likelihood of inaccurate results and this study was therefore excluded. 

In India, a case control study of children aged 0–5 years included 300 hospital cases and 300 community controls involved long and varied periods of recall of breastfeeding details. Univariate results for the ‘use of prelacteal feeds’ and ‘appropriate age of weaning’ are given, without defining exactly what was measured. No results for <12 months given A significant association was found between ARI and lack of breastfeeding, nutritional status, immunization status, delayed weaning, prelactal feeding, living in overcrowded conditions, mothers’ literacy status, low birth weight, and prematurity [19].

No ethics approval was required for this paper as it is a review of previously published studies. Each paper included was a review to ensure that it contained mention of ethics clearance. The results of the studies for respiratory and diarrhoeal diseases were tabulated. It was not possible to undertake a meta-analysis due to the differences in methodology and varying definitions of exposure and outcomes. There were insufficient studies for other infectious conditions to be included in the table, but where adequate information is available, they are mentioned individually.

## 3. Results

The selection process for the 13 studies included in the review produced eight cohort, four cross-sectional, and one case-control study, as detailed in Figure 1.

In almost all of the published studies, breastfeeding was associated with a reduction in the risk of developing an acute respiratory tract infection or diarrhoeal disease. This includes both illness episodes requiring hospital admission or those managed in the community. The reduction in the incidence of these infections is in the order of 50–60% when comparing exclusive or predominant breastfeeding with the use of formula. A number of studies were excluded because of a deficient methodology, but even in those included, standard definitions of breastfeeding were frequently not used. Outcomes, intervals between observations, and definitions varied between studies, making it impossible to undertake a meta-analysis. Nevertheless, the direction of the results showed a strong association between breastfeeding and lower rates of infection in Asia. The studies included in this review are detailed in Table 1.

The study by Yoon in the Philippines used infant deaths as the endpoint. Because of this the numbers were small and showed an effect for deaths due to diarrhoea (24 deaths), diarrhoea and ALRI combined (14 deaths), but did not show an effect of breastfeeding on ALRI by itself (18 deaths) [22].

In a study from Chengdu, China breastfeeding was protective, but if solid foods were introduced before 4 months, the RR of developing a lower respiratory tract infection was 2.996 (1.298–6.916) [20]. This result confirms the importance of the WHO recommendation not to introduce complementary foods before six months of age [34].

The protective effect of breastfeeding on preventing hospital admission for diarrhoeal disease and acute respiratory infection on infants is detailed in Table 1. All of the identified studies showed a protective effect, except one. Typical of the results from a cohort study was an odds ratio for diarrhoeal disease of 0.37 (0.15, 0.88) and 0.39 (0.20, 0.75) for acute respiratory illness [25]. The other studies listed use a number of different methodologies, age ranges, and endpoints, but the direction of the effect is the same. 

### 3.1. Breastfeeding and Specific Infections

Breastfeeding may transmit a number of communicable diseases, including HIV, hepatitis, cytomegalic virus, HTLV1, Ebola, Zika, and the Flavi viruses. Brucellosis has also been reported [35]. The WHO has provided a list of infections where breastfeeding may be contra-indicated or require specific management [36]. However, because of the advantages of breastfeeding, in many cases, particularly in resource-poor communities, the benefits of breastfeeding may outweigh the risks inherent in the use of formula. Infectious conditions included in the WHO advice include: 

Breast abscess: breastfeeding should continue on the unaffected breast; feeding from the affected breast can resume once treatment has started. 

Hepatitis B: infants should be given hepatitis B vaccine, within the first 48 h or as soon as possible thereafter.

Tuberculosis: mother and baby should be managed according to national tuberculosis guidelines [36].

Because of the advantages of breastfeeding, in many cases, particularly in resource poor communities, the benefits of breastfeeding will be greater than the risk of breastfeeding in some of the specific infections included above [36]. While the WHO has given some reasons for not breastfeeding, in some cases in the decade since the publication of these guidelines, advances in treatment have allowed breastfeeding to continue. For example, further information on the transmission through breastmilk and prevalence of HIV, HTLV1, and Zika virus has become available [37].

#### 3.1.1. Measles

WHO has advocated for the elimination of measles through widespread vaccination and public health control measures. Measles vaccination and improved nutrition resulted in a decline in measles deaths of 80% between 2000 and 2017 worldwide. In this period measles vaccination prevented an estimated 21.1 million deaths [38]. However, measles is still a major cause of death in children and there were 110,000 measles deaths globally, with many more infants and children suffering from complications of the disease. In the 12 months to 28 November 2019, the WHO reported 205,000 cases of measles in the SEARO and WPRO regions [39]. 

Recently, there has been a resurgence in measles cases in many countries, including relatively isolated Pacific Islands. Tonga with a population of only 104,000 has had 3728 cases and 53 infant deaths this year [40]. A mass vaccination campaign has been started and 58,000 vaccinated to 1 December 2019. This is reminiscent of the time when Europeans first sailed into the Pacific centuries ago and devastated many islands with measles outbreaks [41]. 

After birth, circulating maternal antibodies provide some protection against measles and this is the basis for the postponement of measles vaccination until later in the first year of life [42]. Breastfeeding provides some protection against measles, but vaccination is required for maximum protection [43]. Early initiation and maintenance of exclusive breastfeeding up until 6 months provides protection for infants with a possible beneficial effect on the vaccine response [44]. After measles infections resistance to other infections decreases often leading to a downward spiral of poor health [45]. Susceptible women who are breastfeeding should be vaccinated with MMR vaccine [46].

#### 3.1.2. Zika Virus

Zika virus infection during pregnancy may result in neurological abnormalities, and the virus and its vector have been reported in the Asian region [47]. The Zika virus can be found in human milk, but a review found no documented studies of long-term complications in infants [48]. The World Health Organization recommends that the benefits of breastfeeding for the infant and mother outweigh any potential risk of Zika virus transmission through breast milk [49].

#### 3.1.3. HIV Infection

In Asia the majority of mothers are tested for STDs, HIV and Hepatitis B during their antenatal visits. The estimated rates of antenatal care are East Asia 96.5%, South East Asia 76.4%, South Asia 50% [50]. Mothers are usually commenced on anti-retroviral therapy (ART) as soon as a diagnosis is made. The use of ART allows breastfeeding to commence and continue with all of the advantages this brings to mother an infant [51]. Breastfeeding should be exclusive until six months before any other fluids or complementary foods are given. This will maintain an optimum microbiome which will contribute resistance to HIV. However, in many Asian countries, if the mother is HIV positive, the infant is started on infant formula after birth as, for example, in Vietnam [52].

#### 3.1.4. Hepatitis B

Hepatitis B remains a common infection that is tested for antenatally in mothers. Despite the high rate of Hepatitis B in Asia, it is estimated that just over one third of infants are vaccinated at birth, despite its proven efficacy [53]. In Hangzhou, China, a cohort study (n = 638) identified 6% of the mothers as being Hepatitis B positive. At one month post-partum, these mothers had a significantly lower rate of ‘any breastfeeding’ compared to non-infected mothers, 62% and 92% respectively, as many had been encouraged by health workers not to breastfeed and to use formula [54]. However, breastfeeding by mothers and infants who have been vaccinated was not associated with HBV infection in the children. Under the recommended prophylaxis, breastfeeding is not a risk factor for mother-to-child transmission of HBV and therefore, clinicians should encourage HBV-infected mothers to breastfeed their infants [55].

#### 3.1.5. Hepatitis C

Globally it is estimated by the WHO that there are 71 million cases of chronic hepatitis C infection world-wide, but other estimates are double this amount [56,57]. Hence, it is not uncommon for a hepatitis C positive mother to breastfeed. Co-infection of hepatitis C and HIV increases the risk of preterm labour [58]. The Center for Disease Control (CDC) recommends that there should be no restrictions on breastfeeding by mothers who are hepatitis C positive [59].

#### 3.1.6. Human T Cell Leukemia Virus (HTLV)

There are an estimated 5–10 million infected persons worldwide infected with HTLV1, and in Asia, infection appears to be more prevalent in Japan and Central Australia [60,61]. It is transmitted by sexual contact, blood transfusions or breastfeeding. The prevalence of the virus is as high as 40% in some outback communities in Australia. While this population has higher rates of some cancers, there is not a large difference in leukemia rates compared to the total Australian population [62]. Other conditions, including chronic renal disease, may also be associated with this infection [60,63]. Where a mother is a known carrier of HTLV, breastfeeding is generally not recommended, but further research is required to establish if this is always the best option.

#### 3.1.7. Helicobacter Pylori

Helicobacter pylori infection remains common throughout Asia and can be acquired at an early age [64]. Reviews of the world-wide distribution of H. pylori sero-positive responses, reported rates from Asia of up to 50% in Malaysia, in Japan 24–29% and in Korea 42% [65,66]. In China, *H pylori* prevalence among symptomatic children fell from 25.6% to 12.8% between 2005 and 2017 [65]. There have been several studies on associations of breastfeeding and Helicobacter infection. The majority of studies support a preventive association between breastfeeding and infection in infancy [67,68]. However, infection rates increase throughout childhood, suggesting that the protective effect of breastfeeding is limited to the early years of life. Further research is required in this area. 

#### 3.1.8. Malaria

Malaria remains an important infection in Asia and can be very difficult to treat in areas where resistance to antimalarial treatment is high. We did not locate any studies from Asia, but studies from Africa suggest that breastfeeding may offer some protection against malaria. In a cross-sectional study from Cameroon (n = 1227), children who had been exclusively breastfed had significantly lower (*P* < 0.001) prevalence of malaria parasite (16.2%) than those not breastfed at all (61.3%) [69]. In a further study from the Democratic Republic of the Congo, 137 EBF infants and 358 non-EBF infants were assessed for fever and malaria infection by polymerase chain reaction (PRC) at six months of age. EBF was associated with a reduced risk of clinical malaria (OR = 0.13; 95% CI = 0.00–0.80), suggesting a protective effect of EBF against malaria [70]. An analysis of stool samples and malaria (PCR) from Mali found that a diverse microbiome was associated with a lower risk of falciparum malaria infection [71]. In the age of chemo-resistance, particularly the increasing rates of artemisinin resistance in Asia, breastfeeding could be important, perhaps as an adjunct to partially effective malaria vaccines. This is a further reason to avoid the unnecessary use of antibiotics and the disruption this causes to the microbiome.

#### 3.1.9. Neonatal Sepsis

A review of infant deaths in Asia found that while overall infant mortality has declined, neonatal infections and deaths have not declined as rapidly and remain a continuing problem [72]. In Bangladesh, neonatal sepsis is the cause of 24% of neonatal deaths, over 65% of which occur in the early-newborn stage (0–6 days). Only 50% of newborns in Bangladesh initiated breastfeeding within 1 h of birth. The early initiation of breastfeeding, within one hour of birth, was associated with the lowest rates of infection [73]. In a meta-analysis of neonatal mortality and breastfeeding, including five out of 10 studies from Asia, the pooled evidence indicated substantial benefits from effective promotion of early initiation of breastfeeding and exclusive breastfeeding during the first month of life [74]. In neonatal intensive care units necrotising enterocolitis (NEC) remains a continuing problem. Wherever possible breastmilk is given to the infant and reduces the incidence of NEC [75,76]. A meta-analysis suggests that unlike breastmilk, donor human milk is not effective in the prevention of NEC [77]. Continuing research is required on effectiveness and mechanisms.

#### 3.1.10. Breastfeeding and Infant Vaccination

Heading the list of the ten greatest public health achievements of the 20th century is “progress in the development of new vaccines and their promotion across the globe”. An example of the powerful nature of vaccination as a public health measure was the elimination of smallpox, a major achievement made possible only by targeted vaccination [78]. Vaccination, along with the promotion of breastfeeding, remains the most important public health intervention against infection in infants and children. There are a number of important public health questions about the relationship between vaccination and breastfeeding: 

Does breastfeeding increase or decrease response to and effectiveness vaccines? 

Does vaccination of mothers prevent illness in infants through the transfer of antibodies and other factors to the infant across the placenta or in breastmilk?

There are a number of biological challenges to to the effective use of, and response to vaccines in lower income countries. Studies on twins show variation in genetics as an important factor to the response to vaccination [79]. Other factors include early life exposure to infection and other vaccines, maternal factors, breastfeeding, age, nutritional status, and other environmental factors [79]. In a review of studies of breastfeeding and vaccinations in infants, Dorea and colleagues found that early breastfeeding initiation, with exclusive breastfeeding to six months, provided maximum protection to infants and a possible beneficial effect on the vaccine responses [44]. Cell-mediated immune response to BCG (Bacille Calmette-Guerin) vaccine given at birth, but not at after one month, was significantly enhanced by breastfeeding. Also, antibody level responses to oral polio vaccine and vaccination with diphtheria and tetanus toxoid were increased in breastfed infants compared to formula-fed. Breastfed infants immunized with Haemophilus influenzae type B, show higher antibody levels at 7 months and 12 months [44]. When vaccines are given by injection breastfeeding provides a significant amount of pain relief and calms the infant [80,81]. More research is needed in Asia on the relationship between breastfeeding and vaccine response. 

#### 3.1.11. Maternal Vaccination

During their antenatal visits, mothers have their vaccination records assessed and are vaccinated where necessary. Maternal vaccination is the only time that vaccination can benefit two generations from a single input, with the possible exception of live polio vaccine [82]. A study in Beijing found that the seroprevalence of maternal and cord antibodies specific for diphtheria, tetanus, pertussis, measles, mumps, and rubella were lower than expected and the authors recommended a vaccination program before pregnancy [83]. Breastfeeding after maternal immunisation during pregnancy has been shown to promote transplacental IgG transfer and after birth, IgA is found in breastmilk. Both mechanisms provide immunological protection to the newborn [84,85]. Some protection from antibodies in breastmilk lasts as long as breastfeeding is continued, but as the infants’ digestive systems develop, denaturation of some antibodies may occur. Other non-specific factors may remain active. Further research is required to assess the extent to which breastmilk antibodies may promote or reduce the effectives of infant vaccination. Influenza vaccine is recommended during pregnancy to protect the newborn and no complications have been reported [86,87,88].

Neonatal tetanus, which is a devastating, infection provides a good case study of the benefits of maternal vaccination. Any health workers who have seen an infant suffering from neonatal tetanus in a low resource setting will never forget the experience. In Papua New Guinea, in the district of Maprik, the infant mortality rate was around 300 with one third due to neonatal tetanus [89]. Implementation of a program of two injections of tetanus toxoid during pregnancy eliminated the problem. However, recently, a shortage of resources and a decline rural health services, has resulted in the disease reappearing [90]. Worldwide, it is estimated that 58,000 neonates from 24 countries still die unnecessarily from the disease every year [91].

#### 3.1.12. Infection from Infant Formula

The review by the US Surgeon General has detailed the increased risk to infants of infection from the use of infant formula instead of breastfeeding [92]. Infant formula may be contaminated by incorrect preparation or storage. In addition, a known contaminant of formula powder is Chronobacter (formerly Enterobactor) *sakazakii.* This bacteria is found primarily in dry and dehydrated foods with low water activity, such as herbal teas, starches, and in powdered infant formula [93]. In formula-fed infants it can occasionally cause infections and fatal neonatal cases have been reported from Japan [94].

## 4. Discussion

This review confirms that the same protective effects of breastfeeding against acute respiratory infection and diarrhoeal disease found in previous reviews in other parts of the world, are also evident in studies from Asia. UNICEF found that the major causes of death worldwide under 12 months of age are pneumonia and diarrhoeal disease [95]. In 2010, diarrhoeal diseases accounted for 11% of the estimated 7.6 million under-five deaths, globally. In Bangladesh, 11% of all under-five deaths (n = 182,936) were due to diarrhea [96]. There was a lower overall rate of diarrhoea in breastfed infants and non-breastfed had a higher rate of rota virus infection in infants 9–12 months [96]. The results for the reduction in deaths by promoting breastfeeding is similar to major reviews from other parts of the world [92,97].

In the WHO regions of East Asia and Pacific, there were 462,000 deaths under the age of five years, including 230,000 neonatal deaths, which was 50% of all under-five deaths. In the WHO South Asia region, there were 1,475,000 deaths under five years and the 909,000 neonatal deaths were 62% of all under five deaths. The population of the Asian countries included in this review was 3.84 billion in 2018. There were 60.3 million births and 1.2 million deaths under one year of age [98]. The weighted infant mortality rate for Asia has declined from 64.5 to 20.1 per 1000 live births between 2000 and 2018. In 2015, it was estimated that improved breastfeeding practices would prevent 823,000 annual deaths in children younger than five years of age and 20,000 annual deaths in women caused by breast cancer [99]. Extrapolating the data from this study, it is estimated that 300,000–350,000 child deaths could be prevented with optimal breastfeeding and the majority would be under 12 months of age.

The exploration of links between nutrition and infection and breastfeeding and infection has progressed during the past six decades, and pioneered the relationship between nutrition and infection. Early workers in the field included Jelliffe, Scrimshaw, and Morley, the latter seeking to emphasise the importance of nutrition to all community health workers [4,100]. In 1986, Ebrahim lamented the decline in breastfeeding worldwide, described breastfeeding as a valuable resource, and gave estimates of value of breastmilk in the USA, Indonesia and Tanzania [101]. He suggested that clinicians and public health workers should be strong advocates for breastfeeding and the control of formula. Since then, much further work has established breastfeeding as the most important link in the nutrition/infection chain of causation. However, despite the importance of breastfeeding being actively promoted by the WHO and UNICEF through research and advocacy, unfortunately, sales of formula continue to increase.

There are numerous bioactive components of breastmilk that contribute to the overall immunological activity of breastmilk, including antibodies, non-specific anti-infective agents, probiotics and white cells, inhibitors of microbiological activity, probiotic bacteria and prebiotic factors, and human cells, leucocytes and lymphocytes [102,103,104,105]. These components of breastmilk provide the mechanism for protection against infection, particularly in the first few months and throughout the breastfeeding period. There is some variation in samples from different parts of the world, although no Asian mothers were included in this study and the relationships with the epidemiology of infection are unclear [106]. Many epidemiological studies and reviews have confirmed the protective role of breastfeeding against infection. [92,97,107,108,109]. 

Once a child contracts an infection, it interferes with nutrition and metabolism, and children are more likely to become undernourished and subsequently to contract further infections [110]. One infection is likely to be followed by another and in the MAL-ED eight-country cohort study, a recent episode of diarrhoea was associated with a higher risk of further episodes of diarrhoea, RR 1.10 (1.04, 1.16), and with higher risk of acute lower respiratory tract infection in the following 3–5-months RR 1.23 (1.03, 1.47) [33].

The microbiome of the infant is important for the development of the immune system, nervous system, and growth [111]. Later in life the microbiome has an important role in reducing chronic disease and preventing obesity [112,113]. Breastfeeding has a very important role in the establishment of a healthy microbiome [114]. If antibiotics are given to treat infections, especially if repeatedly, this can change the microbiome. Dysbiosis of the microbiome is associated with many acute and chronic conditions. 

The role of breastfeeding in protecting against infection has been emphasized in this review. However promoting breastfeeding has also been shown to reduce malnutrition, including poor growth and stunting worldwide [115]. While this review has emphasized the importance of breastfeeding in Asia, the benefits of breastfeeding in reducing infection rates also apply in higher income countries. When summarizing the results of a European cohort study the authors stated that their findings support health policy strategies to promote exclusive breastfeeding for at least 4 months, but preferably six months [12,116].

Despite the overwhelming evidence for breastfeeding offering protection against infection there are increasing sales of infant formula. One of the most important questions related to the implementation of the Sustainable Development Goals is how to increase breastfeeding for all infants [3]. More needs to be done to enforce the standards of the Baby Friendly Hospital Initiative and the WHO Code on the Marketing of Breastmilk Substitutes [108,117,118]. Skin to skin contact between mother and infant within 30–60 min of birth is recommended by UNICEF and WHO and has been shown to improve breastfeeding rates [119]. A review of this practice found considerable variation in the implementation of this practice with rates as low as 9.6% in the Philippines and up to 91% in Singapore [120]. Rates of early contact were much lower in infants delivered by caesarean section in Asia, but the difference was smaller in some Australian studies. Throughout Asia, more effort needs to be given to improve breastfeeding rates as an important step in improving health outcomes and in achieving the Sustainable Development Goals. 

There are several limitations that must be taken into account when considering the results of this review. Ethical restraints have precluded the availability of RCTs and reviews rely only on observational studies. Nevertheless the number of observations increases the quality of the conclusion. In future it may be possible to develop biomarkers or detailed microbiome DNA profiles that would allow ore definitive conclusions. A further constraint is the variability in the definitions used. Very few studies used strict definitions of exclusive breastfeeding.

The lack of randomized controlled trials has meant that the quality of evidence is not ranked highly in the hierarchy of epidemiological studies. However, the sheer number of studies, the lack of any complications from breastfeeding, and the place of breastfeeding means that its importance cannot be understated. Not enough consideration has been given to the risks of introduction of formula or other foods [121]. These risks were well documented in the Surgeon General’s report.

## 5. Conclusions

This review provides details of studies of breastfeeding and infection in Asia. The results show that a minimum 50% reduction of infection rates in infants who are fully breastfed can be expected in the first six months of life. This will result in a significant reduction in costs to the health system and families. The review confirms the results of studies in other regions of the world. There are many shortcomings in studies of breastfeeding, and particular emphasis should be placed on the use of standard definitions. Further, health promotion efforts and environmental change are required to increase the prevalence of breastfeeding.

## Figures and Tables

**Table 1:** Studies of Breastfeeding and Infection in Infants in Asia.

Country	Author	Study Size	Design	Age Months	Breastfeeding Classification	Outcome Measure	Results
Eight Countries incl Nepal India, Bangladesh Pakistan	Richard MALED 2018	1731	Cohort	0–24	EBF compared to ABF	aRR diarr 0–2M	0.58 0.44, 0.76
						aRR Resp	NS
						aRR diarr 3–5M	0.83 0.75, 0.93
						aRR Resp	0.81 0.68, 0.98
Maldives	Raheem 2017	458	Cohort	0–6	Predominant BF 6/12 Y = 153, N = 305	ARTI aOR	0.45 (0.24–0.84)
						Diarrhoea aOR	0.31 (0.10–0.90)
China (urban)	Yu [20] 2016	682	Cohort	0–6	Any BF 1/12 (Y = 607 N = 75)	aOR LRTI (<6/12)	0.479(0.263-0.872)
Vietnam (rural)	Hanieh 2015	1049	Cohort	0–6	Exclusive BF at 6 weeks (32.8%)	Diarrhoea OR	0.37 (0.15 to 0.88)
						Pneumonia OR	0.39 (0.20,0.75)
India Rural	Panda 2014	696	Cohort	0–6	EBF compared to ABF	aOR diarr	0.49 (0.27, 0.90)
Bangladesh Rural	Mihrshahi 2008	351	Cohort	0–6	EBF compared to Partial BF	aOR diarr	0.29 (0.12, 0.68
						aOR ARI	0.4 (0.21, 0.75)
Bangladesh Urban	Arifeen 2001	1677	Cohort	0–12	Predominant breastfeeding compared to partial or none	All deaths aHR	0.45 (0.29, 0.69)
						ARI deaths	0.42(0.20, 0.88)
						Diarrhoea	0.25 (0.09,0.68)
Philippines	Yoon 1996	9942	Cohort	0–12	Not BF compared to Breastfed Death rates 0–5 months	aRR diarr	0.10 (0.25,0.04)
						aRR ALRI	NS
						aRR ALRI	0.17 (0.56–0.05)
Philippines	Hengstermann 2010	399	Case control	0–6	Risk of hospitalisation Exclusive breastfeeding & Formula fed	Any Infection aOR	0.29 (0.17,0.48)
						Diarrhoea aOR	0.05 (0.02,0.15)
						Pneumonia aOR	0.36 (0.19,0.66)
China (rural)	Li 2019 [21]	1802	Cross sect	6–12	Any BF = 1049 Not BF = 753) Illness in past month	Diarrhoea *p* < 0.01	BF 33%, No BF 42%
						Cough *p* = 0.03	BF 43% NoBF49%
Bangladesh national	Khan 2017	1918	DHS cross sectional	0–6	EBF T 0–2 M	aOR diarr	0.20 (0.10, 0.32)
						aOR ARI	0.42 (0.31, 0.79)
					EBF T 2–4 M	aOR diarr	0.32 (0.20, 0.47
						aOR ARI	0.71 (0.57, 0.90)
					EBF T 4–6 M	aOR diarr	0.43 (0.31, 0.53)
						aOR ARI	0.84 (0.64, 0.96)
China (urban)	Cai 2016	1654	Cross sect	0–12	Exclusive BF Mixed Exc Formula	Hospitalisation EBF compared to Exc Formula	Respiratory illness
							OR 0.69 (0.50, 0.96)
Bangladesh Rural	Mihrshahi 2007	1633 DHS	cross section	0–3	EBF 0–3 M compared other	aOR diarr	0.69 (0.49–0.98)
						aOR ARI	0.69 (0.54–0.88)

IMR = infant mortality rate, HR = hazard ratio, aOR = adjusted odds ratio, ARI = acute respiratory infection, M = months, DHS = Demographic and Health Survey. ALRI = acute lower respiratory infection, diarrh = diarrhoeal diseae, EBF = Exclusive breastfeeding, EFF = Exclusive formula feeding, PBF = Predominant breastfeeding, ABF = Any breastfeeding, * = *P* < 0.05. Note in the Arifeen study deaths <4 days of age were not included to exclude birth trauma and fatal congenital abnormalities. In the Khan study ‘T’ indicates that Exclusive breastfeeding was terminated 2, 4 or 6 months as indicated. The references used in compiling Table 1 are: [20,21,22,23,24,25,26,27,28,29,30,31,32,33].
